# Online monitoring applying the anaerobic respiratory monitoring system reveals iron(II) limitation in YTF medium for *Clostridium ljungdahlii*

**DOI:** 10.1002/elsc.202000054

**Published:** 2020-11-05

**Authors:** Marcel Mann, Darina Wittke, Jochen Büchs

**Affiliations:** ^1^ AVT – Biochemical Engineering RWTH Aachen University Aachen Germany

**Keywords:** acetogens, *C. ljungdahlii*, carbon dioxide transfer rate, medium optimization, online monitoring

## Abstract

Online monitoring of microbial cultures is an effective approach for studying both aerobic and anaerobic microorganisms. Especially in small‐scale cultivations, several parallel online monitored experiments can generate a detailed understanding of the cultivation, compared to a situation where a few data points are generated from time course sampling and offline analysis. However, the availability of small‐scale online monitoring devices for acetogenic organisms is limited. In this study, the previously reported anaerobic Respiration Activity MOnitoring System (anaRAMOS) device was adapted for online monitoring of *Clostridium ljungdahlii* (*C. ljungdahlii*) cultures with fructose as the carbon source. The anaRAMOS was applied to identify conversion of different carbon sources present in commonly used YTF medium. An iron(II) deficiency was discovered in this medium for *C. ljungdahlii*. Addition of iron(II) to the YTF medium reduced the cultivation time and increased biomass yield of *C. ljungdahlii* cultures by 50% and 40%, respectively. The measurement of the carbon dioxide transfer rate was used to calculated the iron(II) contained in complex components. By demonstrating the application of the anaRAMOS device for medium optimization, it is proven that the described online monitoring device has potential for use in process development.

## 1 INTRODUCTION

The cultivation of organisms capable of utilizing gaseous carbon and energy sources has been intensively studied in recent years [1, 2, 3, 4]. Fermentation using gaseous carbon sources is highly flexible with respect to the origins of the gas. For example, industrial off‐gases, gasified lignocellulosic biomass, or gasified municipal waste can be used as substrates [4, 5, 6, 7]. Microbial fermentation of gaseous carbon sources, which commonly consist of a mixture of carbon monoxide (CO), carbon dioxide (CO_2_), and hydrogen (H_2_) (syngas), is an alternative process to Fischer–Tropsch synthesis [8]. Syngas fermentation is less affected by gaseous impurities, while being more flexible with respect to the gas composition. Owing to lower temperature and pressure requirements compared to chemical processes, the overall energy consumption for heating and pressurizing is lower in syngas fermentations [1, 9]. Furthermore, a range of molecules, from acetate and ethanol to longer chain alcohols and acids, can be produced using syngas fermentation [3, 10, 11, 12].

The most commonly studied microorganism for syngas fermentation is *Clostridium ljungdahlii* (*C. ljungdahlii*), a strict anaerobic spore‐forming acetogen capable of metabolizing carbon monoxide and carbon dioxide in combination with hydrogen, which was first isolated in 1988 [13]. Besides gaseous carbon and energy sources, *C. ljungdahlii* is able to metabolize several solid carbon and energy sources, such as C_5_ and C_6_ sugars [14]. However, *C. ljungdahlii* growth on solid carbon sources is rarely reported in literature, although it is useful for medium optimization and growth characterization. Different medium compositions are used to culture *C. ljungdahlii*. While the ATCC 1754 medium is mainly used in studies investigating gaseous carbon sources, YTF medium with fructose as the main carbon source is commonly used in studies focusing on genetic engineering of *C. ljungdahlii* [15, 16, 17, 18].

Acetogenic organisms, such as *C. ljungdahlii*, are mainly cultured in serum bottles at small scales [11, 15, 19], or in lab‐scale bioreactors such as stirred tank reactors (STR) or bubble column reactors. Serum bottles are used for screening multiple conditions simultaneously. However, offline sampling intervenes with cultivation and alters the culture conditions. Usually, samples are taken from the same serum bottles throughout one experiment, instead of using one serum bottle per sampling point. Furthermore, culture conditions in a closed system, such as serum bottles, change throughout the cultivation period due to gas production and consumption, affecting the gas composition and pressure.

The availability of small‐scale online monitoring devices, which are widely used for aerobic processes [20], are currently rather limited for anaerobic processes. Bioreactors, on the other hand, provide several online monitoring tools and maintain constant culture conditions throughout the experiment via constant aeration, pH control, etc. However, performing several bioreactor experiments simultaneously for screening purposes is laborious and expensive. The recently developed anaerobic respiration activity monitoring system (anaRAMOS) is the first device combining online monitoring with semi‐continuous ventilation in small‐scale cultivation of anaerobic organisms [21]. The AnaRAMOS holds up to eight 250 mL shake flasks that are semi‐continuous ventilated with nitrogen, while being shaken at a constant temperature. Carbon dioxide sensors allow the measurement of the carbon dioxide concentration in the head space, thereby enabling the calculation of the carbon dioxide transfer rate (CTR). Generally, the measured CTR is assumed to be equal to the carbon dioxide evolution rate; thus, representing a direct measure of the metabolic activity, as reported for aerobic processes [22].

This study aims to validate the use of the anaRAMOS device for monitoring the cultivation of the model acetogen *C. ljungdahlii* with fructose as the main carbon source. By demonstrating the optimization of the commonly used YTF medium, the potential and advantages of the anaRAMOS device for future research are demonstrated.

**PRACTICAL APPLICATION**

The designed to measure the carbon dioxide transfer rate in shake flasks, was adapted and used to monitor the growth of the model acetogen *Clostridium ljungdahlii* (*C. ljungdahlii*). In this study, an iron(II) limitation in the commonly used YTF medium for *C. ljungdahlii* was determined using the anaRAMOS. Cultivation in YTF medium with added iron(II) reduced the cultivation time by 50% and increased the biomass yield by 40% compared to that in YTF without added iron(II). By combining the online monitoring device with the presented experimental approach, a fast, reliable, and easy process optimization and the discovery of medium deficiencies can be achieved.

## 2 MATERIAL AND METHODS

### 2.1 Strain and medium

*C. ljungdahlii* (DSM 13528) was kindly provided by Fraunhofer IME (Germany) as an actively growing culture in YTF medium in a 250 mL serum bottle. Initial cryo stocks were stored as 2 mL aliquots with 10% anoxic DMSO at ‐80°C. YTF medium containing 10 g yeast extract (Yeast extract, Oxoid™, France, Lot: 2522291‐02), 16 g tryptone (Tryptone/Peptone aus Casein, Roth, Germany, Lot: 395234974), 4 g NaCl, 0.75 g cysteine‐HCl, and 5 g fructose per liter was used for all cultivations. A 1.5‐fold medium stock solution containing yeast extract, tryptone, and NaCl was prepared and autoclaved at 121°C for 20 min. Sterile filtered cysteine‐HCl and autoclaved fructose from a 20‐fold concentrated stock solution were added prior to each experiment. The pH was set to 6 using 1 M HCl and the final volume was adjusted using autoclaved deionized water. The medium was transferred into serum bottles and RAMOS flasks under sterile conditions. Serum bottles were ventilated for 20 min using ultra‐high purity (99.999%) nitrogen (Praxair, Germany). Subsequently, 10 mL (for 100 mL serum bottles) or 25 mL (for 250 mL serum bottles) carbon dioxide (99.995%) (Praxair, Germany) was added to each serum bottle using a syringe and needle. For anaRAMOS cultivations, RAMOS flasks containing aerobic medium were installed in the anaRAMOS device and semi‐continuously ventilated using ultra‐high purity (99.999%) nitrogen for a minimum of 2 h to completely remove oxygen from the gas phase and dissolved oxygen from the medium.

### 2.2 Cultivation conditions

Two consecutive pre‐cultures were performed for each experiment. The first pre‐culture with a total volume of 10 mL (in 100 mL serum bottles) contained 1 mL of thawed cryo stock and was cultivated for 24 h. The second pre‐culture with a total volume of 50 mL (in 250 mL serum bottles) was inoculated in a 1:10 ratio. After 20–24 h, an optical density of 1.0 was reached and the second pre‐culture was terminated. The main cultures were inoculated in a 1:10 ratio, unless stated otherwise. Inoculation took place after an initial ventilation phase of the anaRAMOS device for a minimum of 2 h by transferring pre‐culture through a septum into the RAMOS flasks using a 5 mL syringe with a needle (0.6 mm × 30 mm). For the experiment shown in Figure 2, the second pre‐culture was, in contrast to the other experiments, grown in the anaRAMOS (Supplementary Figure S8). The main cultures were inoculated using a 5 mL syringe with a needle (0.6 mm × 30 mm) immediately after the CTR dropped to 0 mmol L^–1^ h^‐1^.

All main cultures were performed in unbaffled 250 mL shake flasks at 37°C in an orbital shaker (climo shaker ISFX‐1, Kühner AG, Switzerland) using an in‐house built anaRAMOS device according to Munch et al. 2019 [21]. A filling volume (V_L_) of 50 mL was used at a shaking diameter (d_0_) of 50 mm and a shaking frequency (n) of 100 rpm. The anaRAMOS device was used to semi‐continuously monitor the carbon dioxide transfer rate in up to eight parallel shake flasks. Each flask was equipped with an inlet and an outlet valve (Supplementary Figure S1A positions 2 and 6), a pressure sensor (Type 26PCA, Honeywell Inc., USA) (Supplementary Figure S1A position 3), a micro pump (Microfluidic piezo membrane pump, Bartels Mikrotechnik, Germany) (Supplementary Figure S1A position 4), and a CO_2_ sensor (MSH P CO_2_, Dynament, UK) (Supplementary Figure S1A position 5). The pump (4) continuously circulated gas from the head space past the CO_2_ sensor (5). The direction of the gas flow is shown in Supplementary Figure S1A. Measurement of the CTR includes three phases: the flow phase (1), the measurement phase (2), and the high flow phase (3) depicted in Supplementary Figure S1B. During the flow phase, each shake flask was individually ventilated using ultra‐high‐purity (99.999%) nitrogen (Praxair, Germany) at a flow rate of 5 mL min^–1^ per shake flask. During the measurement phase, the inlet and outlet valves allowed closure of in‐gas and off‐gas, resulting in a closed gas head space during the measurement phase. The linear increase in the carbon dioxide concentration, monitored by the CO_2_ sensor within the measurement phase, was used to calculate the CTR [21]. Prior to each experiment, a pressure test was performed with pressure sensors located within the gas loop to ensure a fully air‐tight system. Further details on the setup and calculation methods have been reported previously [21].

### 2.3 Offline analysis

For offline analysis, 3 mL of the sample was removed from one shake flask using a 5 mL syringe with a needle (0.6 mm × 30 mm). Sampled shake flasks were not further considered for online and offline analyses, as cultivation conditions are altered due to volume changes. The optical density (OD) was measured after appropriate dilution with sterile medium at a wavelength of 600 nm using a Photometer (Genesys 20 Photospectrometer, ThermoSpectronic, USA). The optical density was used to calculate the carbon content of the biomass. Therefore, the OD to BTM correlation in Equation 1 was used (compare to Supplementary Figure S2).

(1) OD=0.0019·CDW

A biomass composition of CH_1.666_N_0.23_O_0.27_ with a molecular weight of 20.7 g mol^–1^ was assumed [23]. The remaining sample was centrifuged for 5 min at RCF = 18 000 × g. A portion of the supernatant was used for pH measurement (HI221 Microprocessor pH Meter, Hanna Instruments, Germany) and the remainder was stored at –20°C for high‐performance liquid chromatography (HPLC) analysis. For HPLC analysis, samples were thawed, filtered (pore size 0.2 μm), and stored at 4°C until analysis. HPLC (Prominence HPLC, Shimadzu, Germany) was performed using an organic acid column (ROA‐Organic Acid H+, Phenomenex Inc., Germany) at 60°C and a flow rate of 0.8 mL min^–1^ of 5 mM H_2_SO_4_. Elution was detected using a refractive index detector (RID‐10A, Shimadzu, Germany).

## 3 RESULTS AND DISCUSSION

### 3.1 Adaptation of *C. ljungdahlii* to cultivation conditions

The anaRAMOS was used for monitoring the cultivation of *C. ljungdahlii* in YTF medium. A previous study has reported the use of anaRAMOS with a gas ventilation rate of 10 mL min^–1^ N_2_ per flask for the cultivation of *Clostridium pasteurianum* (*C. pasteurianum*) [21]. Since the metabolic activity of *C. ljungdahlii* is lower than that of *C. pasteurianum*, the ventilation rate was reduced to 5 mL min^–1^ N_2_, decreasing CO_2_ removal from the flasks because CO_2_ is beneficial for anaerobic organisms [24].

### 3.2 Reference culture

**FIGURE 1:**
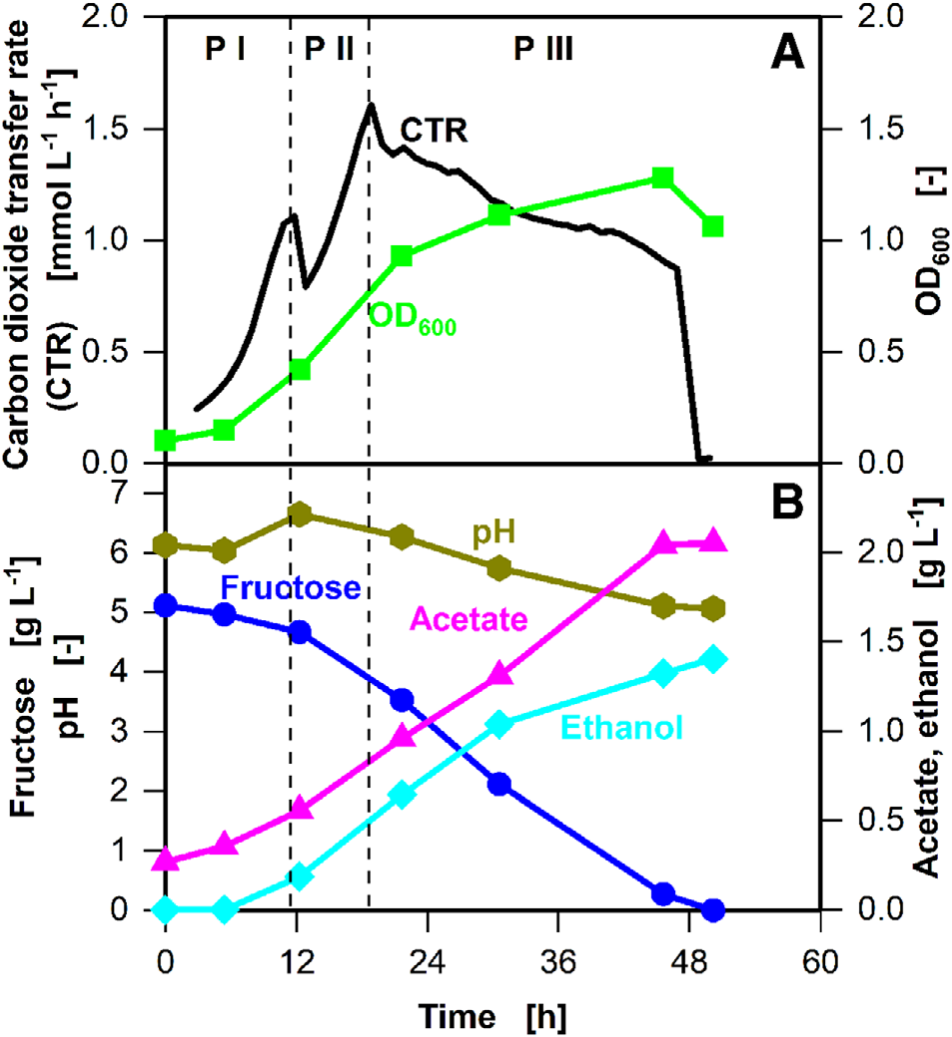
Parallel batch cultivation of *C. ljungdahlii* DSM 13528 in seven 250 mL shake flasks containing YTF medium. One online shake flask was removed for each sampling time point. P I, P II, and P III indicating phase one, phase two, and phase three of the cultivation are defined in Supplementary Figure S3. (A) Carbon dioxide transfer rate (CTR) and optical density at 600 nm wavelength (OD_600_) (B) Fructose, acetate, and ethanol concentrations. Cultivation conditions: Inoculation density OD_ _= 0.1, inoculation from actively growing pre‐culture in serum bottle, temperature (T) = 37°C, pH = 6, shaking frequency n = 100 rpm, shaking diameter d_0_ = 50 mm, filling volume V_L_ = 50 mL, initial fructose concentration c = 5 g L^–1^, ventilation using 100% N_2_, and flow rate q_in_ = 5 mL min^–1^. The data that support the findings of this study are openly available at [DOI: 10.18154/RWTH‐2020‐10467]

To establish a reference cultivation, *C. ljungdahlii* was cultivated in YTF medium containing 5 g L^‐1^ fructose (Figure 1). For better understanding, the cultivation is divided into three phases, as indicated in Figure 1 and clarified in Supplementary Figure S3. To validate the CTR measured online, offline samples were regularly taken and the sampled flasks were not used for further analysis. The comparability among the different parallel flasks in one experiment is shown in Supplementary Figure S4. Variations were observed in the reference cultures of different cultivations (Supplementary Figure S5). This likely results from slight variations in the pre‐cultures. Pre‐culture has been previously reported as a major factor impacting microbial cultivation [25].

The CTR indicated exponential growth for approximately 12 h (Phase I), reaching a CTR of approximately 1.1 mmol L^‐1^ h^–1^ (logarithmic plot is shown in Supplementary Figure S6). During this period, the OD (Figure 1A) increased to 0.4, pH increased slightly, and fructose concentration decreased. The increase in pH is likely due to the consumption of complex components, as previously reported for other organisms [26, 27]. The product concentration (ethanol and acetate) increased by 0.4 g L^–1^. While no ethanol was produced in the pre‐culture, a slight acetate carryover into the main culture was observed (Figure 1B).

After 12 h, the CTR shows an intermittent drop before further exponentially increasing to a maximum value of 1.6 mmol L^‐1^ h^‐1^ after 20 h (Phase II) (Logarithmic plot is shown in Supplementary Figure S6). The OD during the second growth phase increased further to 1.0 at the maximum CTR (after 20 h). At the same time, the pH decreased due to increasing acetate concentration; ethanol concentration increased similarly. The decrease in CTR after phase I as well as the different growth rates represented by the slopes of the logarithmic CTR plots (μ_max_ = 0.084 h^–1^ versus μ_max_ = 0.053 h^–1^, compare to Supplementary Figure S6) are indicators of a metabolic switch, which has previously been denoted as diauxic effect for aerobic cultures. After 20 h (start of Phase III), the CTR slowly starts to decrease and reaches a value of 0.9 mmol L^‐1^ h^‐1^ after 48 h. Subsequently, the CTR drops to 0 mmol L^‐1^ h^‐1^. During phase III, the OD increases at a lower rate compared to cultivation phases I and II, while fructose concentration decreases steadily and depletes at the same time as the CTR drops to 0 mmol L^‐1^ h^‐1^. The pH during phase III decreased and reached a minimum value of 5.1 after 48 h. The product concentration further increased to maximum values of 2 g L^–1^ for acetate and 1.5 g L^–1^ for ethanol. The slightly decreasing plateau of the CTR during phase III is most likely the result of a secondary substrate limitation, as reported in case of aerobic cultivations [28, 29]. The ODs measured offline throughout the cultivation indicate that the growth of *C. ljungdahlii* was similar to data published elsewhere [17].

The initial fructose concentration as well as the acetate and biomass carryovers from the pre‐culture can be used to calculate the amount of carbon initially added to the cultivation. In total, 183 mmol_Carbon_ L^‐1^ was added at the start of the cultivation (excluding the amount of carbon added via complex components). An equivalent amount of carbon is expected in the products (carbon dioxide, biomass, acetate, and ethanol). The integral of the CTR curve from phases I–III can be used to calculate the total carbon dioxide evolution (Supplementary Figure S3). Based on HPLC results, the total amount of carbon in ethanol and acetate can be calculated, and the OD can be used to calculate the total carbon in the biomass. At the end of the cultivation, a total amount of 200 mmol_Carbon_ L^–1^ was measured in the products, including ethanol, acetate, biomass, and carbon dioxide. A detailed distribution of carbon between the substrates and products is plotted in Supplementary Figure S7. The deviation between the amounts of carbon in the substrates and products is most probably the result of the undefined amount of carbon added via complex components.

### 3.3 Identification of the cultivation phases observed in reference cultures

**FIGURE 2:**
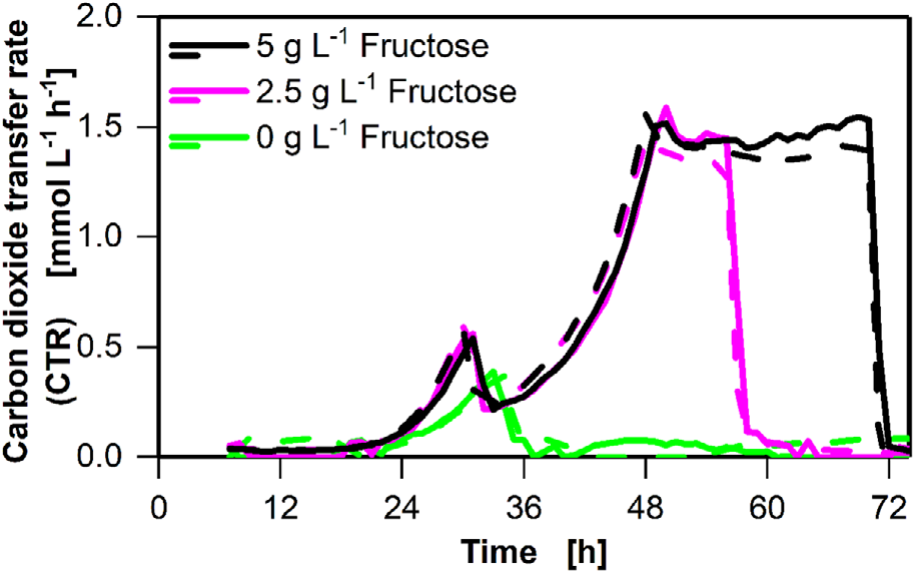
Variation of initial fructose concentrations in batch cultivations of *C. ljungdahlii* DSM 13528 in six 250 mL shake flasks. Fructose concentration varied between 0, 2.5, and 5 g L^–1^. Carbon dioxide transfer rate (CTR), duplicates are indicated by dashed lines. Cultivation conditions: Inoculation from cryogenic culture, YTF medium, temperature T = 37°C, pH = 6, shaking frequency n = 100 rpm, shaking diameter d_0_ = 50 mm, filling volume V_L_ = 50 mL, initial fructose concentration c = 5 g L^–1^, ventilation using 100% N_2_, and flow rate q_in_ = 5 mL min^–1^. The data that support the findings of this study are openly available at [DOI: 10.18154/RWTH‐2020‐10467]

After establishing a reference cultivation as described above, it was attempted to identify the different phases observed during cultivation on 5 g L^–1^ fructose (Figure 2). Therefore, different initial fructose concentrations (0, 2.5, and 5 g L^–1^) were added to the subsequent cultures (Figure 2). All cultivations (0, 2.5, and 5 g L^–1^ of fructose) shown in Figure 2 were inoculated from a pre‐culture grown in anaRAMOS immediately after carbon source depletion (pre‐culture progression is plotted in Supplementary Figure S8), preventing fructose carryover from the pre‐culture into the main culture. Inoculation from stationary phase cultures resulted in a prolonged lag phase of approximately 18 h, which was not observed in cultivations inoculated from actively growing cultures (Supplementary Figure S5).

After a lag phase of approximately 24 h, phase I of all cultivations was comparable to that of the previous cultivation (Figure 2) (Figure 1A). Since growth in phase I was also observed for the cultivation with 0 g L^‐1^ fructose, it may be deduced that the growth was necessarily based on complex components added via yeast extract and tryptone. Phase II was not observed for the cultivation with 0 g L^–1^ fructose. The cultivations with 2.5 and the 5 g L^–1^ fructose were run in parallel for phase II. The duration of phase III depended on the fructose concentration. In phase III, 2.5 g L^‐1^ of fructose depleted after 58 h, whereas 5 g L^–1^ fructose depleted after 71 h. The plot in Figure 2 clearly identifies the diauxic growth. During phase I, complex components are utilized; thereafter, the additional carbon source fructose is consumed.

**FIGURE 3:**
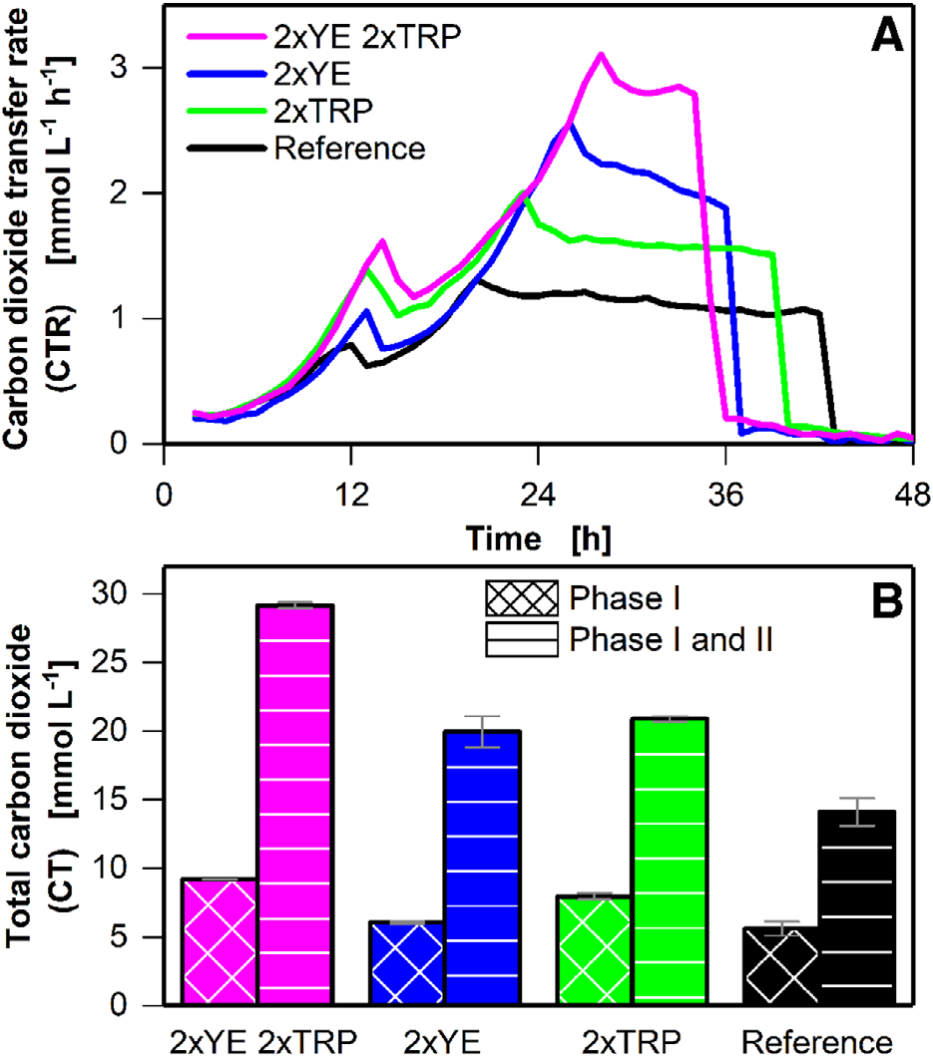
Influence of complex components on batch cultivation of *C. ljungdahlii* (DSM 13528) in eight 250 mL shake flasks. Concentrations of complex components varied among flasks, reference cultures in standard YTF medium, and cultivation with either doubled yeast extract (2xYE) concentration or doubled tryptone (2xTRP) concentration or both doubled yeast extract and double tryptone (2xYE; 2xTRP) concentrations. (A) Carbon dioxide transfer rate (CTR). Duplicates shown in Supplementary Figure S9 (B) Accumulated carbon dioxide (CT) calculated on the basis of the online CTR measurement for phase I and phase I + II, as defined in Supplementary Figure S3. Error bars indicate minimal and maximal values. Cultivation conditions: Inoculation density OD_ _= 0.1, inoculation from actively growing pre‐culture in serum bottle, YTF medium, temperature T = 37°C, pH = 6, shaking frequency n = 100 rpm, shaking diameter d_0_ = 50 mm, filling volume V_L_ = 50 mL, initial fructose concentration c = 5 g L^–1^, ventilation using 100% N_2_, and flow rate q_in_ = 5 mL min^–1^. The data that support the findings of this study are openly available at [DOI: 10.18154/RWTH‐2020‐10467]

To investigate the influence of the complex components in cultivations, higher concentrations of these components (yeast extract or/and tryptone) were used (Figure 3), while fructose concentration was maintained at 5 g L^–1^. With increasing concentrations of complex components, the duration of phase I increased (Figure 3A). While phase I for the reference cultivation is 12 hours, doubled yeast extract as well as doubled tryptone lead to a phase I duration of 13 hours. Interestingly, the maximum CTR in the first peak for cultivation with doubled yeast extract concentration increased by 0.2 mmol L^–1^ h^–1^ compared to the reference, whereas doubled tryptone concentration led to an increase in the first peak by 0.5 mmol L^–1^ h^–1^ compared to the reference cultivation. Thus, tryptone promotes higher metabolic activity during phase I compared to yeast extract. This is also demonstrated by the higher amount of carbon dioxide produced during the first peak when the tryptone concentration was increased compared to that when the yeast extract concentration was increased (Figure 3B). When the concentrations of both yeast extract and tryptone were doubled in one cultivation, phase I was prolonged to 14 h and a maximum CTR of 1.6 mmol L^‐1^ h^‐1^ was reached. This represents the sum of the maximum CTR of the reference (in phase I) and the increase in the CTR when yeast extract and tryptone were added separately (0.9 + 0.2 + 0.5 = 1.6 mmol L^‐1^ h^‐1^).

As expected, the duration of phase II until reaching the plateau increased with an increase in the amounts of complex components added. However, plateau formation in phase III was still observed in all cultivations. Doubled yeast extract concentration increased the duration of phase II from 7 to 11 h, whereas doubled tryptone concentration increased this duration from 7 to 9 h. Hence, doubled yeast extract increased phase II duration by 4 h, while doubled tryptone concentration increased this duration by 2 h. Based on the increase in duration when only tryptone concentration or yeast extract concentration was increased, the expected increase when both tryptone and yeast extract are doubled can be calculated. The expected increase from 7 to 13 h (2 h increase (tryptone) + 4 h increase (yeast extract) = 6 increase) was observed in the experimental results (Figure 3A). The same trend was observed for the amount of carbon dioxide produced during phase I + II (Figure 3B). The more the amounts of complex components added, the higher is the amount of carbon dioxide produced during phase I + II. Hence, the secondary substrate limitation indicated by the plateau formation occurs later, with the addition of more complex components.

Yeast extract and tryptone are rich in proteins, amino acids, and other organic supplements. These compounds are likely not limiting. Therefore, macro or trace elements may limit cultivation. Available literature suggest iron(II) dependency of *C. ljungdahlii* [30] and other acetogenic *Clostridia* such as *C. carboxydivorans* [31] and *C. ragsdalei* [32], and hence, the influence of iron(II) addition was exemplarily tested to demonstrate the potential of the anaRAMOS for process development.

### 3.4 Iron(II) supplementation for unlimited growth

**FIGURE 4:**
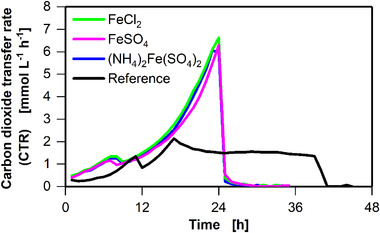
Influence of different iron(II) compounds on the carbon dioxide transfer rate (CTR) of batch cultivations of *C. ljungdahlii* (DSM 13528) in four 250 mL shake flasks. 8 mg L^–1^ ammonium iron(II) sulfate hexahydrate ((NH_4_)_2_Fe(SO_4_)_2_·6H_2_O), 2.58 mg L^‐1^ of iron(II) chloride (FeCl_2_), or 5.67 mg L^–1^ iron(II) sulfate (FeSO_4_) was added to the cultivation, resulting in 20 μmol L^–1^ iron(II). Cultivation conditions: Inoculation density OD_ _= 0.1, inoculation from actively growing pre‐culture in serum bottle, YTF medium, temperature T = 37°C, pH = 6, shaking frequency n = 100 rpm, shaking diameter d_0_ = 50 mm, filling volume V_L_ = 50 mL, initial fructose concentration c = 5 g L^–1^, ventilation using 100% N_2_, and flow rate q_in_ 5 = mL min^‐1^. The data that support the findings of this study are openly available at [DOI: 10.18154/RWTH‐2020‐10467]

To overcome the secondary substrate limitation anticipated based on the experiments described earlier, iron(II) was tested as a potential candidate in the subsequent experiments. In Figure 4, the effect of three different iron(II) compounds on the cultivation of *C. ljungdahlii* in YTF medium is demonstrated. Of these, FeSO_4_ and FeCl_2_ were chosen to ensure that neither ammonium nor sulfate introduced via ammonium iron(II) sulfate influence the cultivation conditions.

As no reference culture was performed for the variations of the iron(II) compounds, the reference of another experiment is plotted in Figure 4. The observed difference in phase I likely results from slight differences in the pre‐cultures. As shown in Supplementary Figure S5, the CTR progression of the reference cultivations varies slightly among the different experiments. Apart from the difference in CTR progression, phase I was observed as in all previous experiments, despite the addition of iron (II).

The addition of 20 μmol L^–1^ iron(II) (in the form of FeCl_2_, FeSO_4_, and (NH_4_)_2_Fe(SO_4_)_2_) resulted in exponential growth in phase II, without the formation of a plateau (Figure 4), as observed in previous experiments (Figures 1, 2, 3). In the reference culture, phase III started after approximately 16 h. Phase III was not observed at all when the CTR dropped after 24 h, and fructose was depleted. Compared to the reference culture, the maximum CTR reached 6.5 mmol L^‐1^ h^‐1^ when iron(II) was added.

**FIGURE 5:**
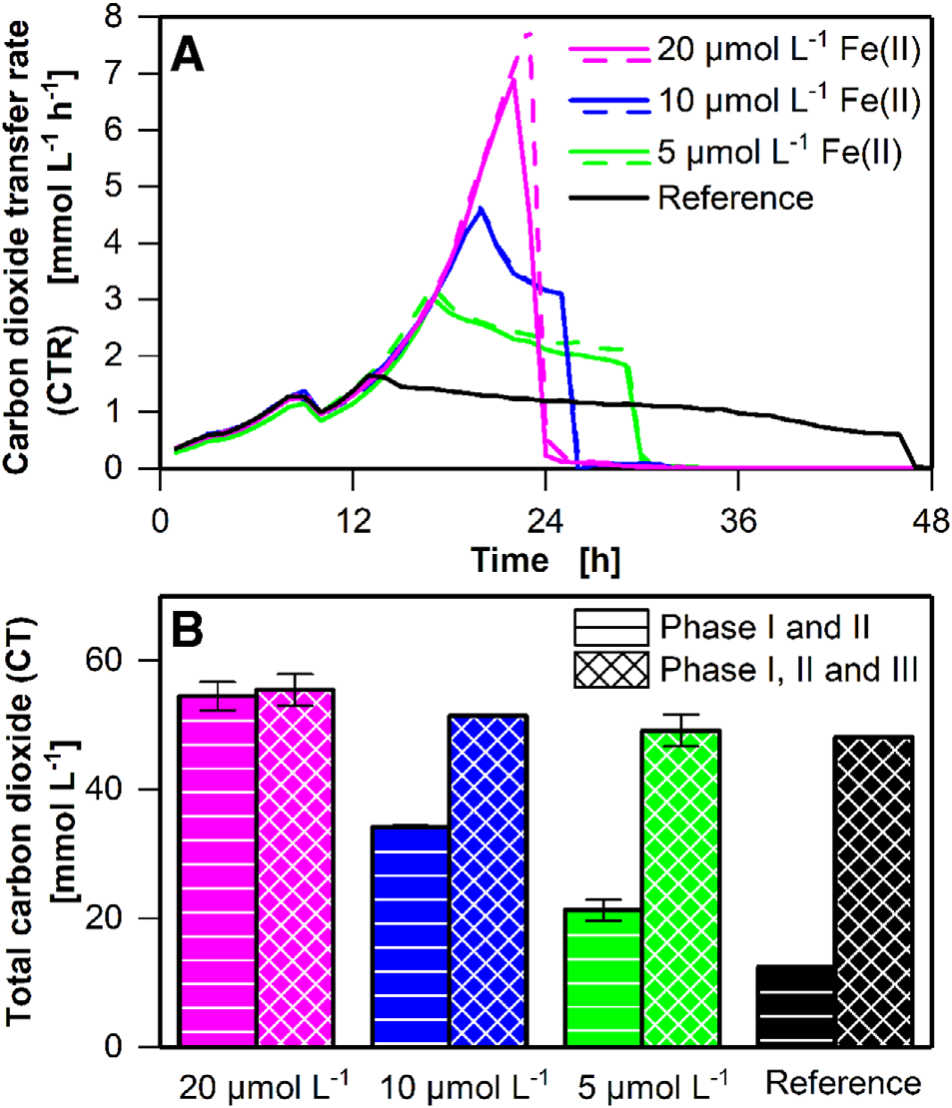
Influence of the available amounts of iron(II) on batch cultivation of *C. ljungdahlii* (DSM 13528) in seven 250 mL shake flasks. Iron(II) concentration added via ammonium iron(II) sulfate hexahydrate ((NH_4_)_2_Fe(SO_4_)_2_·6H_2_O) varied among cultivations with 0, 5, 10, and 20 μmol L^–1^ fructose. (A) Carbon dioxide transfer rate (CTR), duplicates are indicated by dashed lines. (B) Accumulated carbon dioxide (CT) calculated based on the online CTR measurement. Error bars indicate minimal and maximal values. Cultivation conditions: Inoculation density OD_ _= 0.1, inoculation from actively growing pre‐culture in serum bottle, YTF medium, temperature T = 37°C, pH = 6, shaking frequency n = 100 rpm, shaking diameter d_0_ = 50 mm, filling volume V_L_ = 50 mL, initial fructose concentration c = 5 g L^–1^, ventilation using 100% N_2_, and flow rate q_in_ = 5 mL min^–1^. The data that support the findings of this study are openly available at [DOI: 10.18154/RWTH‐2020‐10467]

As three different iron(II) compounds consistently resulted in unlimited growth (Figure 4), iron(II) can certainly be identified as the limiting component in the reference culture. To determine the necessary amount of iron(II), different iron(II) concentrations were added to *C. ljungdahlii* cultures in YTF medium, as shown in Figure 5. As iron(II) concentration increased, phase II duration also increased, whereas phase III duration decreased. The addition of 20 μmol L^–1^ iron(II) led to unlimited growth (phase III was not observed as discussed for Figure 4 earlier), with a maximum CTR of 7.5 mmol L^–1^ h^–1^.

Figure 5B shows the degree of limitation based on the carbon dioxide exhaust. For unlimited growth following the addition of 20 μmol L^–1^ of iron(II), the total carbon dioxide (CT) for the whole cultivation (Phases I, II, and III) was equal to the CT until iron(II) becomes limiting (Phase I and II) (Compare to Supplementary Figure S3). When the amount of added iron(II) was reduced, the CT until iron(II) becomes limiting (Phase I and II) decreased, while the CT of the complete cultivation (Phase I, II, and III) decreased only slightly. The slight decrase in CT of the whole cultivation (Phase I, II, and III) with decreasing amounts of iron(II) results from the conversion of C_6_ compounds into three C_2_ compounds without the production of CO_2_ by *C. ljungdahlii* [33]. As the cultviation duration increases with less iron(II) added, more CO_2_ can be converted. CO_2_ converted into soluble products (acetate, ethanol, or biomass) cannot be detected by the anaRAMOS. An increase in the iron(II) concentration results in faster growth. Thus, the same amount of CO_2_ as in the reference culture is produced in a shorter period of time, resulting in higher CO_2_ concentration in the head space of the shake flask. Hence, more CO_2_ was removed from the shake flask before being utilized by *C. ljungdahlii*.

**FIGURE 6:**
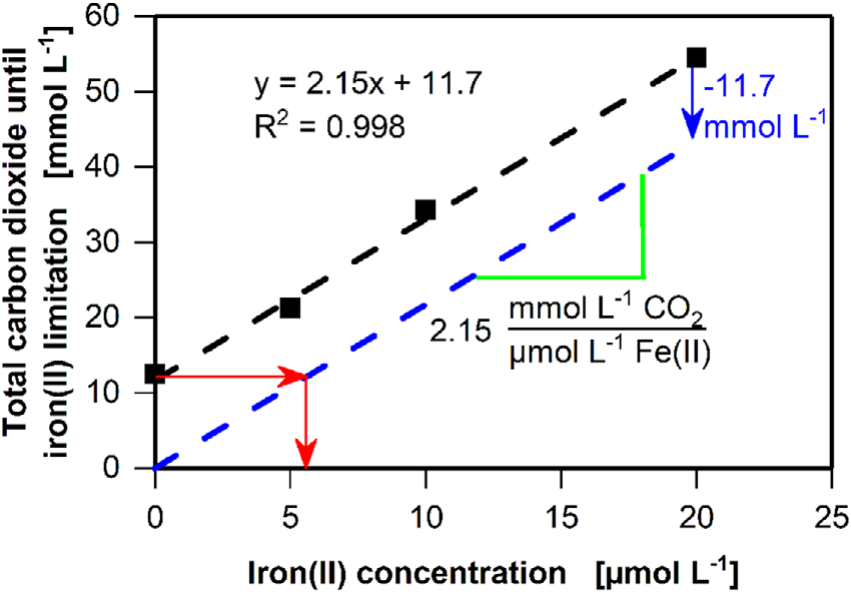
Total carbon dioxide until iron(II) limitation (Phase I and II) plotted against the iron(II) concentration based on experimental data shown in Figure 5. Black squares indicate measured values from Figure 5B (crosshatched bars). Black line indicates correlation. Blue line indicates the total carbon dioxide production resulting from iron(II) additionally supplemented to the medium in the form of ammonium iron(II) sulfate hexahydrate ((NH_4_)_2_Fe(SO_4_)_2_·6H_2_O). Green triangle indicates the dependence of the total carbon dioxide until iron(II) limitation based on the iron(II) concentration. Red lines indicate the concentration of iron(II) initially added via complex components

The relationship between the amount of CO_2_ produced until iron(II) becomes limiting (Phase I and II) (compare to Supplementary Figure S3) and the iron(II) concentration added to the medium is plotted in Figure 6. Therefore, the CT of phase I + II shown in Figure 5B with a known amount of iron(II) is used. Without the addition of iron(II), 11.7 mmol L^–1^ CO_2_ was produced in phase I+II, representing the iron(II) added via the complex components (yeast extract and tryptone) of the YTF medium (Figure 6). As shown in Figure 6, 11.7 mmol L^–1^ of the carbon dioxide produced could be traced back to approximately 5.5 μmol L^‐1^ iron(II) added to the reference culture in YTF medium via complex components. Furthermore, it can be calculated that 2.15 mmol L^–1^ CO_2_ was produced per μmol L^–1^ iron(II) under unlimited conditions. These findings can be used to adapt the iron(II) concentration to the amount of carbon source provided. During glycolysis, the pathway used for fructose conversion, one molecule of fructose is converted into two molecules of CO_2_ and two molecules of acetyl‐CoA. Hence, 5 g L^–1^ of fructose resulted in approximately 55 mmol L^‐1^ CO_2_ production, theoretically. Based on this information, it can be calculated that 11 mmol CO_2_ was produced per 1 g of fructose. As shown in Figure 6, 25.1 μmol L^–1^ iron(II) was needed for the unlimited production of 55 mmol L^–1^ CO_2_, which was equivalent to the conversion of 5 g L^–1^ of fructose. In total, 25.5 μmol L^–1^ iron(II) (5.5 μmol L^–1^ iron(II) from complex components, and 20 μmol L^–1^ iron(II) in the form of ammonium iron(II) sulfate hexahydrate) was added to the unlimited cultivation shown in Figure 5.

**FIGURE 7:**
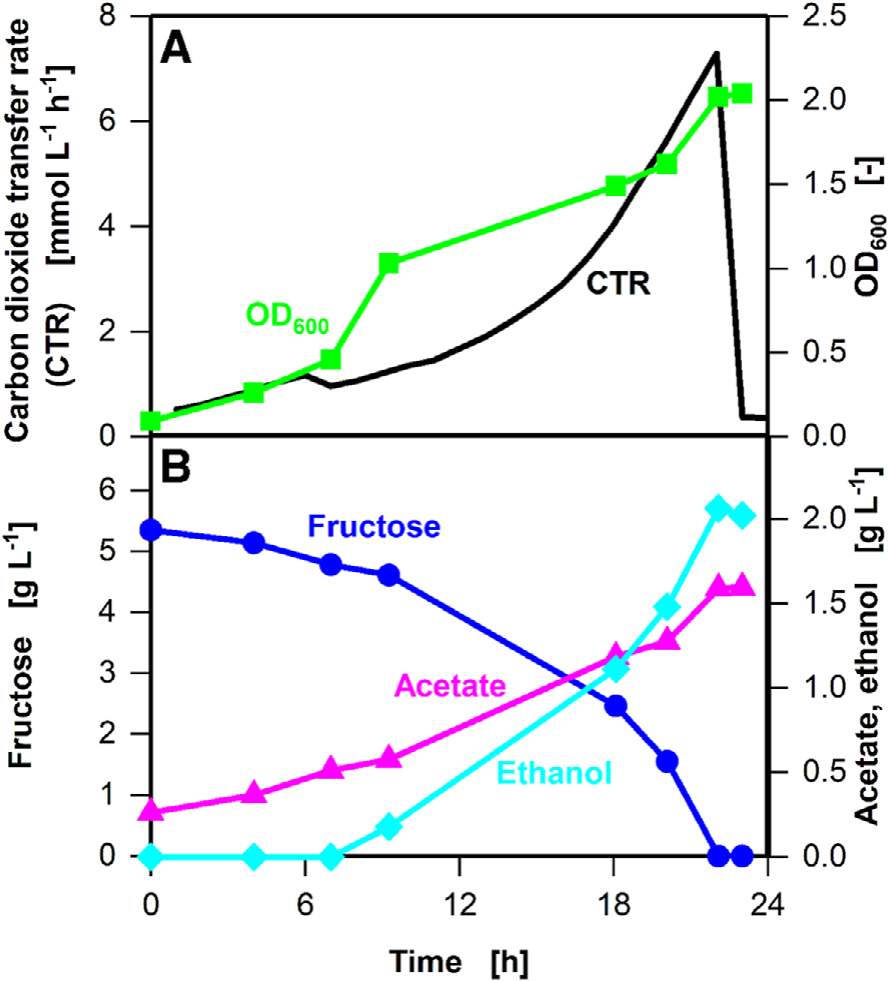
Optimized parallel batch cultivation of *C. ljungdahlii* DSM 13528 in eight 250 mL shake flasks containing YTF medium with the addition of 8 mg L^–1^ ammonium iron(II) sulphate hexahydrate ((NH_4_)_2_Fe(SO_4_)_2_·6H_2_O) (resulting in 20 μmol L^–1^ iron(II)). One online shake flask was removed for each sampling time point. (A) Carbon dioxide transfer rate (CTR) and optical density (OD) (B) Fructose, acetate, and ethanol concentrations. Cultivation conditions: Inoculation density OD_ _= 0.1, inoculation from actively growing pre‐culture in serum bottle, temperature T = 37°C, pH = 6, shaking frequency n = 100 rpm, shaking diameter d_0_ = 50 mm, filling volume V_L_ = 50 mL, initial fructose concentration c = 5 g L^–1^, ventilation using 100% N_2_, and flow rate q_in_ = 5 mL min^–1^. The data that support the findings of this study are openly available at [DOI: 10.18154/RWTH‐2020‐10467]

To further validate the results and confirm the data generated by online monitoring of the CTR, cultivation was performed using the optimized medium composition. Offline sampling of a cultivation with 20 μmol L^–1^ iron(II) (Figure 7) supported the findings previously described on the basis of the online signals shown in Figures 4 and 5. The CTR plotted in Figure 7A shows the same progression as described for Figures 4 and 5A. The final optical density after 22 h of cultivation was 2.0 (Figure 7A), compared to 1.2 after 48 h in standard YTF medium (Figure 1A). Thus, the addition of iron(II) resulted in a 40% increase in the biomass concentration compared to that without added iron(II). The cultivation time (phase I – III) in medium containing added iron(II) was reduced by 50% compared to that in medium without added iron(II). When iron(II) was added, the product spectrum shifted slightly from acetate to ethanol (2.0 vs. 1.6 g L^–1^ of acetate and 1.4 vs. 2.0 g L^‐1^ of ethanol) (Figure 7B and Figure 1B). This shift maybe due to an iron(II) ‐ dependent alcohol dehydrogenase present in *C. ljungdahlii* [30]. The results plotted in Figure 7 clearly show a distinct influence of the addition of iron(II).

## 4 CONCLUSION

The aim of this study was to establish the cultivation of the acetogenic model organism *C. ljungdahlii* using anaRAMOS and to improve the composition of the commonly used YTF medium. AnaRAMOS, as described previously [21], proved to be a useful online measurement tool for monitoring the cultivation of the acetogenic model organism *C. ljungdahlii*. AnaRAMOS allows for online monitoring in semi‐continuously ventilated shake flasks. However, its application has not been previously reported for acetogenic organisms. Online monitoring of the CTR revealed iron(II) limitation in the commonly used YTF medium for *C. ljungdahlii*. The addition of iron(II) led to a 3‐fold increase in the maximum CTR, 40% increase in biomass, and a 50% reduction in cultivation time compared to cultivation in YTF medium without added iron(II).

## CONFLICT OF INTEREST

The authors have declared no conflict of interest.

## Supporting information

Supporting information
