# Supplementary material for: Online monitoring applying the anaerobic respiratory monitoring system reveals iron(II) limitation in YTF medium for Clostridium ljungdahlii
Source: Eng Life Sci. 2020 Nov 5;21(1-2):19–28. doi: 10.1002/elsc.202000054 (PMC7837299; doi:10.1002/elsc.202000054)
Supplement: Supplementary file 1 — Supporting information [file ELSC-21-19-s001.pdf]

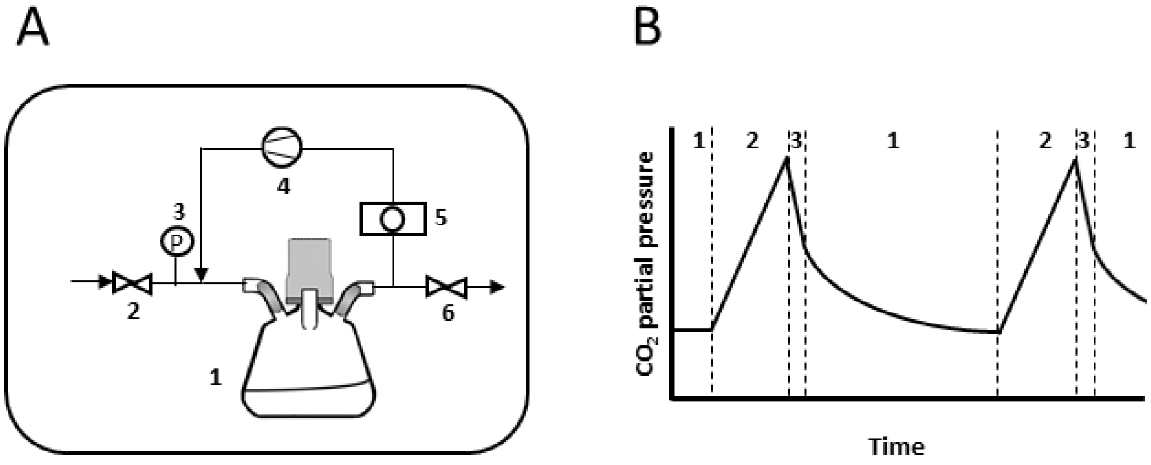

Supplementary Figure 1: Schematic setup of one out of eight anaRAMOS measurement positions. **A:** Shows the flask setup with details of gas flows, valve and sensor position. **1)** Single RAMOS flask containing a microbial culture; **2)** Inlet valve, open during flow phase, closed during measurement phase; **3)** Differential pressure sensor for pressure; **4)** Circulation pump, conveying gas from the headspace of the RAMOS flask to the carbon dioxide sensor (5) and back to the flask at a flow rate of 20 mL/min tests; **5)** Carbon dioxide sensor for online monitoring of the carbon dioxide partial pressure; **6)** Outlet valve, open during flow phase, closed during measurement phase. **B**: Typical progression of CO_2 partial pressure during a cultivation on fructose. 1**)** Flow phase (40 minutes) with open inlet and outlet valve for gas flow through the headspace of the anaRAMOS. **2)** Measurement phase (20 minutes) with closed inlet and outlet valve, 3) High flow phase (1 minute) with 8- fold increase of the gas flow rate.

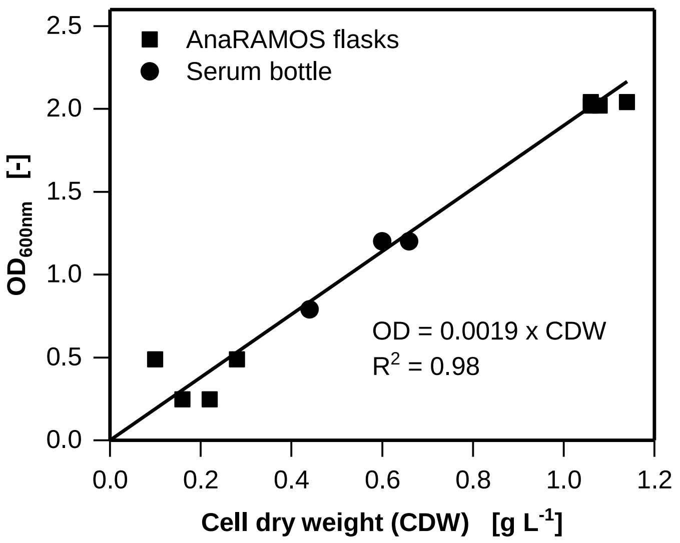

Supplementary Figure 2: Correlation of the optical density (OD_{600}) [-] and the cell dry weight (CDW) [g L^{-1}] of *C. ljungdahlii* (DSM 13528). Samples were taken from parallel cultivation in serum bottles (circles) and the anaRAMOS (squares). Cultivation conditions serum bottles: Inoculation density OD = 0.1, inoculation from actively growing pre-culture in serum bottle, YTF medium, temperature T = 37°C, pH = 6, shaking frequency n = 100 rpm, shaking diameter d_0 = 50 mm, filling volume V_L = 40 mL in 250 mL serum bottles, initial fructose concentration c = 5 g L^{-1}, 100 % N_2 gas. Cultivation conditions anaRAMOS: Inoculation density OD = 0.1, inoculation from actively growing pre-culture in serum bottle, YTF medium, temperature T = 37°C, pH = 6, shaking frequency n = 100 rpm, shaking diameter d_0 = 50 mm, filling volume V_L = 50 mL, initial fructose concentration c = 5 g L^{-1}, ventilation using 100 % N_2, flow rate q_{in} = 5 mL min^{-1}.

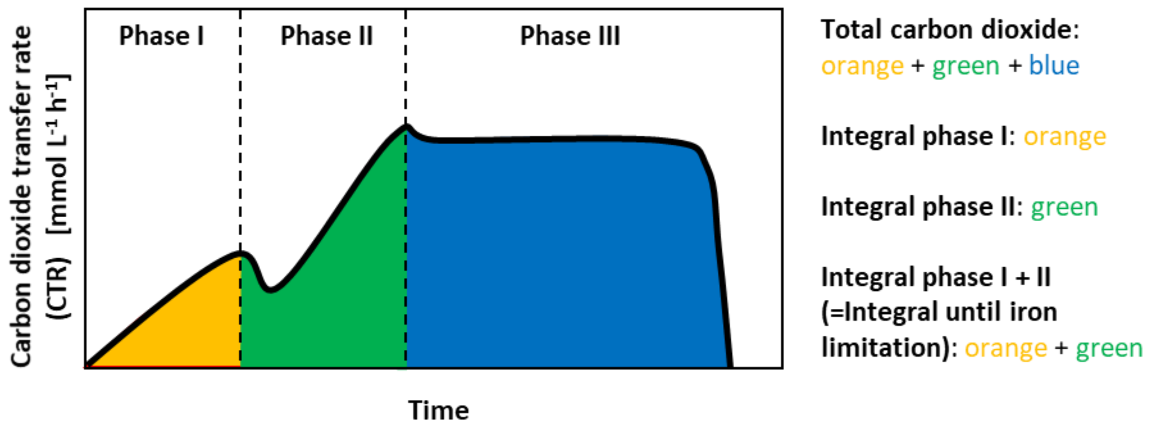

Supplementary Figure 3: Schematic outline and definition of the different cultivation phases and the respective integrals.

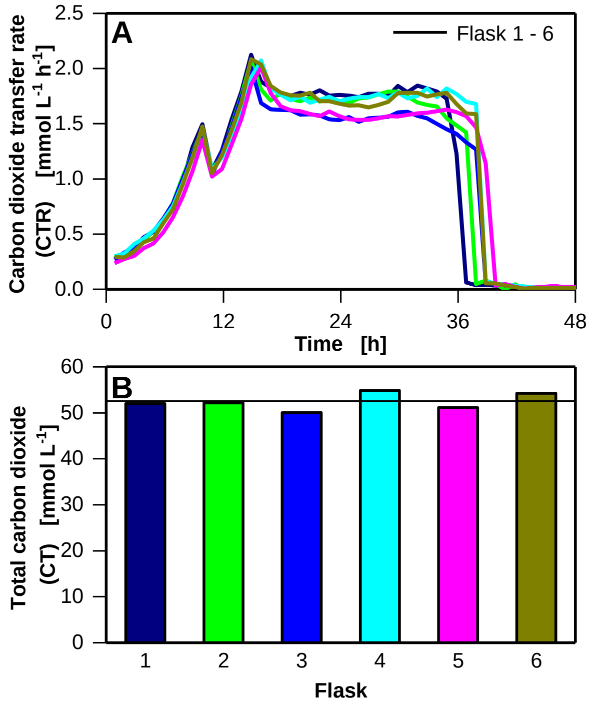

Supplementary Figure 4: Comparison of identically performed reference cultivations of *C. ljungdahlii* (DSM 13528) in six parallel 250 mL shake flasks. **A:** Carbon dioxide transfer rate (CTR) **B:** Accumulated carbon dioxide (CT) calculated from the online CTR measurement for phases I + II + III. Straight line at 52 mmol L^{-1} indicates expected total carbon dioxide production. Cultivation conditions: Inoculation density OD = 0.1, inoculation from actively growing pre-culture in serum bottle, YTF medium, temperature T = 37°C, pH = 6, shaking frequency n = 100 rpm, shaking diameter d_0 = 50 mm, filling volume V_L = 50 mL, initial fructose concentration c = 5 g L^{-1}, ventilation using 100 % N_2, flow rate q_{in} = 5 mL min^{-1}.

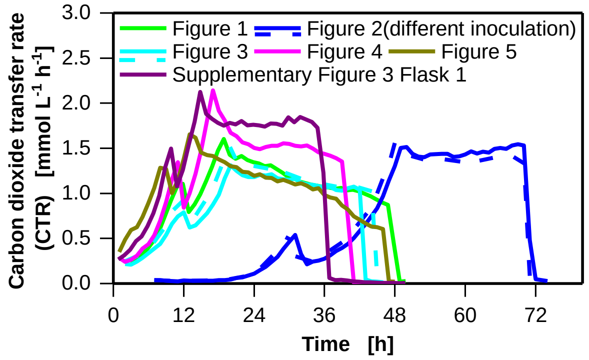

Supplementary Figure 5: Reference cultivations of experiments plotted in Figures 1-5 and Supplementary Figure 3, Flask 1. Cultivation curve Figure 2 resulting from a different pre-culture procedure. Duplicates are indicated by dashed lines. Cultivation conditions: Inoculation density OD = 0.1, inoculation from actively growing pre-culture in serum bottle, YTF medium, temperature T = 37°C, pH = 6, shaking frequency n = 100 rpm, shaking diameter d_0 = 50 mm, filling volume V_L = 50 mL, initial fructose concentration c = 5 g L^{-1}, ventilation using 100 % N_2, flow rate q_{in} = 5 mL min^{-1}.

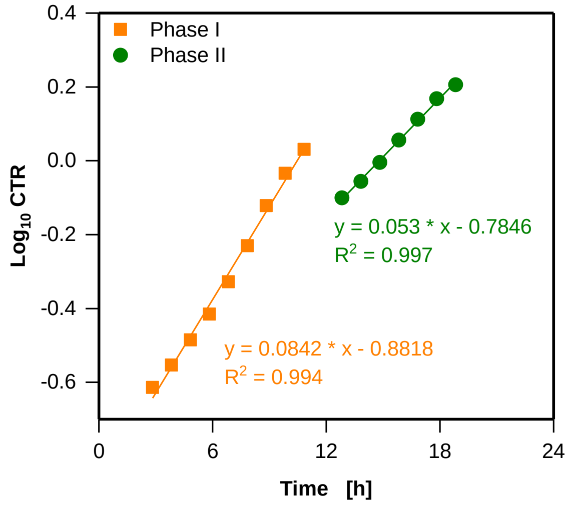

Supplementary Figure 6: Logarithmic plot of the carbon dioxide transfer rate (CTR) shown in Figure 1. The values for phase I and phase II are shown in different colors. Cultivation conditions: Inoculation density OD = 0.1, inoculation from actively growing pre-culture in serum bottle, temperature T = 37°C, pH = 6, shaking frequency n = 100 rpm, shaking diameter d_0 = 50 mm, filling volume V_L = 50 mL, initial fructose concentration c = 5 g L^{-1}, ventilation using 100 % N_2, flow rate q_{in} = 5 mL min^{-1}.

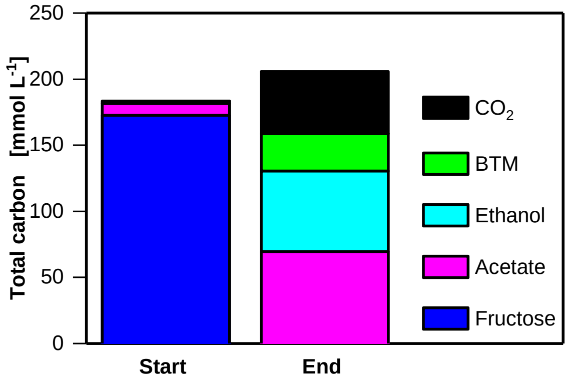

Supplementary Figure 7: Carbon in substrate (start) and carbon in products (end) of the cultivation plotted in Figure 1. Cultivation conditions: Inoculation density OD = 0.1, inoculation from actively growing pre-culture in serum bottle, YTF medium, temperature T = 37°C, pH = 6, shaking frequency n = 100 rpm, shaking diameter d_0 = 50 mm, filling volume V_L = 50 mL, initial fructose concentration c = 5 g L^{-1}, ventilation using 100 % N_2, flow rate q_{in} = 5 mL min^{-1}.

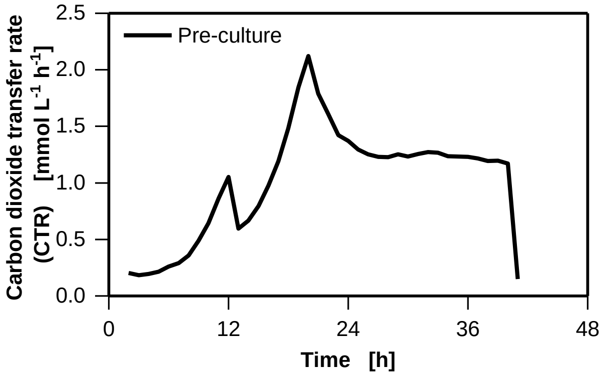

Supplementary Figure 8: Pre-culture for the experiment depicted in Figure 3. *C. ljungdahlii* (DSM 13528) was cultivated in one 250 mL shake flasks and used as a pre-culture after the CTR declined due to fructose depletion (41 h). Cultivation conditions: Inoculation density OD = 0.1, inoculation from actively growing pre-culture in serum bottle, YTF medium, temperature T = 37°C, pH = 6, shaking frequency n = 100 rpm, shaking diameter d_0 = 50 mm, filling volume V_L = 50 mL, initial fructose concentration c = 5 g L^{-1}, ventilation using 100 % N_2, flow rate q_{in} = 5 mL min^{-1}.

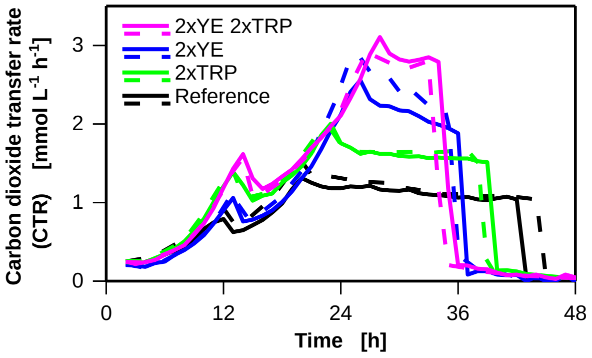

Supplementary Figure 9: Duplicates for Figure 3A. Influence of complex components on batch cultivation of *C. ljungdahlii* (DSM 13528) in eight 250 mL shake flasks. Concentration of complex components varied between each flask, reference with standard YTF medium, double yeast extract (2xYE), double tryptone (2xTRP), double yeast extract and double tryptone (2xYE 2xTRP). Carbon dioxide transfer rate (CTR), duplicates are indicated by dashed lines. Cultivation conditions: Inoculation density OD = 0.1, inoculation from actively growing pre-culture in serum bottle, YTF medium, temperature T = 37°C, pH = 6, shaking frequency n = 100 rpm, shaking diameter d_0 = 50 mm, filling volume V_L = 50 mL, initial fructose concentration c = 5 g L^{-1}, ventilation using 100 % N_2, flow rate q_{in} 5 = mL min^{-1}.

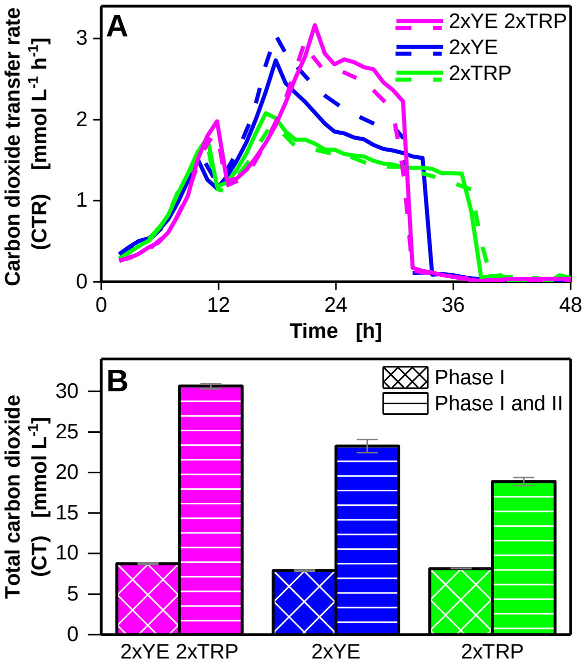

Supplementary Figure 10: Repetition of experiment shown in Figure 3. Influence of complex components on batch cultivation of *C. ljungdahlii* (DSM 13528) in six 250 mL shake flasks. Concentration of complex components varies between each flask. Double yeast extract (2xYE), double tryptone (2xTRP), double yeast extract and double tryptone (2xYE 2xTRP). **A:** Carbon dioxide transfer rate (CTR), duplicates are indicated by dashed lines. **B:** Accumulated carbon dioxide (CT) calculated from the online CTR measurement for phase I, and phase I + II, as illustrated in Supplementary Figure 2. Error bars indicate minimal and maximal values. Cultivation conditions: Inoculation density OD = 0.1, inoculation from actively growing pre-culture in serum bottle, YTF medium, temperature T = 37°C, pH = 6, shaking frequency n = 100 rpm, shaking diameter d_0 = 50 mm, filling volume V_L = 50 mL, initial fructose concentration c = 5 g L^{-1}, ventilation using 100 % N_2, flow rate q_{in} = 5 mL min^{-1}.
